# First report of euglena-induced anaphylaxis: A case study

**DOI:** 10.5415/apallergy.0000000000000208

**Published:** 2025-12-02

**Authors:** Emi Murakami, Masataka Suehiro, Wataru Sasaki, Rina Kamigaki, Akio Tanaka, Kaori Ishii

**Affiliations:** 1 Department of Dermatology, Graduate School of Biomedical and Health Sciences, Hiroshima University, Minami-ku, Hiroshima, Japan

**Keywords:** Anaphylaxis, immediate allergy, euglena

## Abstract

Euglena, a microalga with photosynthetic capabilities, has become increasingly popular in Japan as a health food and supplement owing to its rich nutrient profile. However, their potential to cause allergic reactions remains largely unknown. We report the first case of anaphylaxis induced by euglena in a 48-year-old Japanese woman with a history of urticaria and oral allergy syndrome. She had been consuming a Euglena-containing supplement for nearly a decade, increasing her intake in the 3 months preceding her initial visit. She experienced recurrent anaphylactic episodes, including urticaria, abdominal pain, palpitations, and vomiting. Diagnostic tests, including histamine release and skin-prick tests, confirmed an immediate allergic reaction to Euglena. Following discontinuation of the Euglena-containing supplement, her symptoms resolved completely. As the consumption of Euglena-based products continues to increase, the possibility of Euglena-induced allergic reactions should be considered, and further research into its allergenic components is necessary.

## 1. Introduction

*Euglena* (Japanese name: Midorimushi) is a microalga with chloroplasts that are capable of photosynthesis. Owing to its rich nutrient content and the development of cultivation technologies, *Euglena* has gained significant attention in Japan for use in health foods and supplements [1]. This report describes the first known case of anaphylaxis induced by *Euglena*-based products in a Japanese woman, diagnosed through histamine release tests (HRT) and skin-prick tests.

## 2. Case presentation

A 48-year-old female patient presented with a 5-year history of urticaria. Approximately 3 months before her initial visit, she experienced systemic urticaria and vomiting, requiring emergency transport. Following this episode, she experienced 4 additional anaphylactic symptoms, which included combinations of urticaria with abdominal pain, urticaria with abdominal pain and palpitations, and abdominal pain with palpitations and hand swelling. Owing to the recurrence of these anaphylactic symptoms, she was referred to our department for further examination and treatment.

Her medical history included a uterine fibroid and a history of oral allergy syndrome triggered by apple, cherry, kiwi, loquat, persimmon, and pear. Upon reviewing her medications, she regularly consumed a *Euglena*-containing supplement. She had been taking this supplement for nearly a decade, initially consuming 1 tablet every few days and increasing to 2–3 tablets every other day at dinner for the 3 months preceding her initial visit. Allergic symptoms were induced about an hour after oral ingestion of the tablet containing Euglena. There were no clear co-factors such as exercise or alcohol consumption in symptom induction.

**Figure 1.**
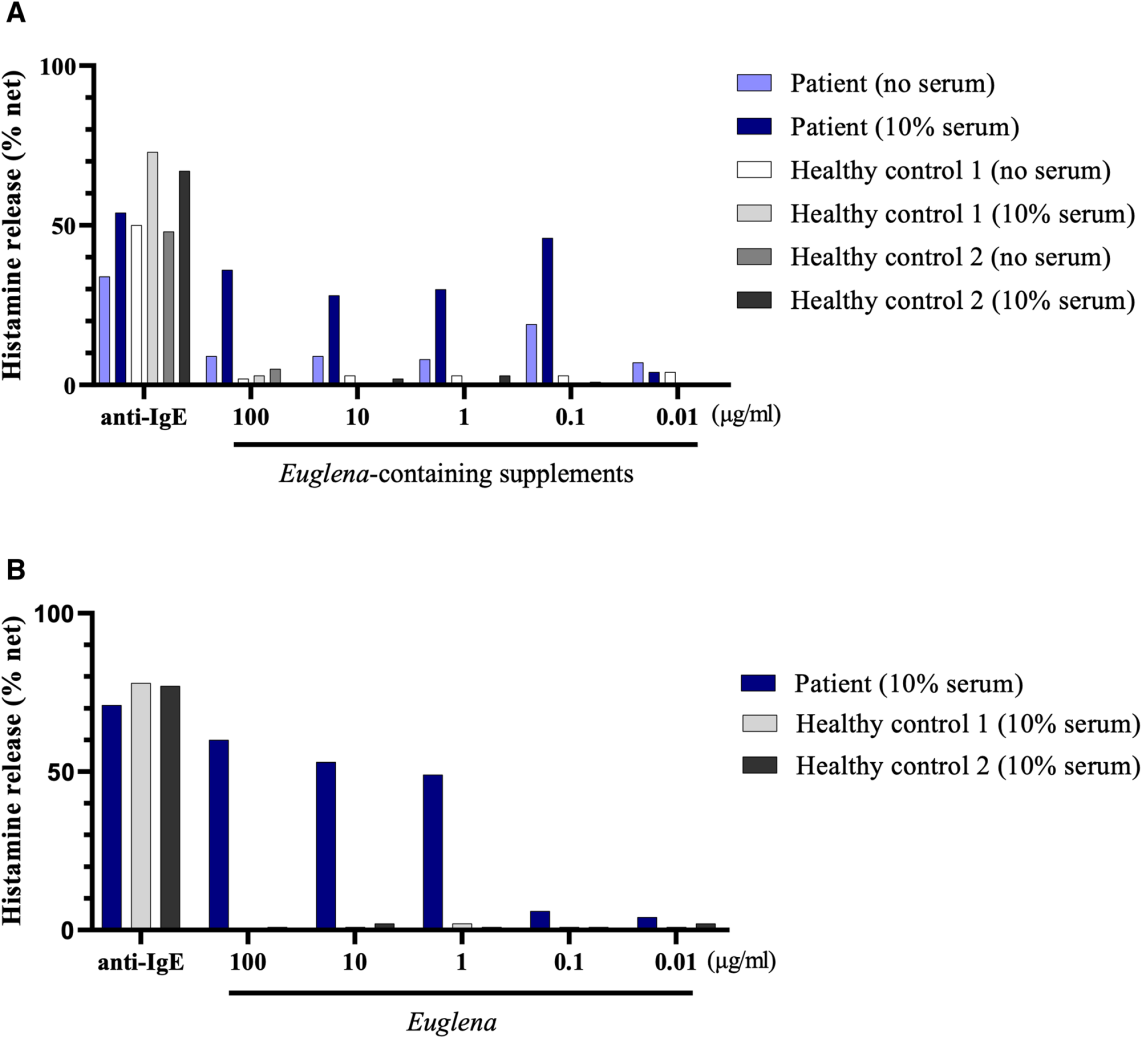
Histamine release in response to Euglena-containing supplements and raw materials. **(A**) Histamine release test (HRT) for *Euglena*-Containing Supplements. Histamine release from the patient’s basophils was induced by the *Euglena*-containing supplement, while no histamine release was observed in healthy controls. The addition of 10% autologous serum to the patient’s sample enhanced histamine release, aiming to more closely mimic the in vivo response. **(B**) HRT for *Euglena*. Among the individual raw materials tested, only *Euglena*-induced histamine release from basophils at concentrations of 1 μg/ml or higher. No histamine release was observed with any of the other raw materials (data not shown).

**Figure 2.**
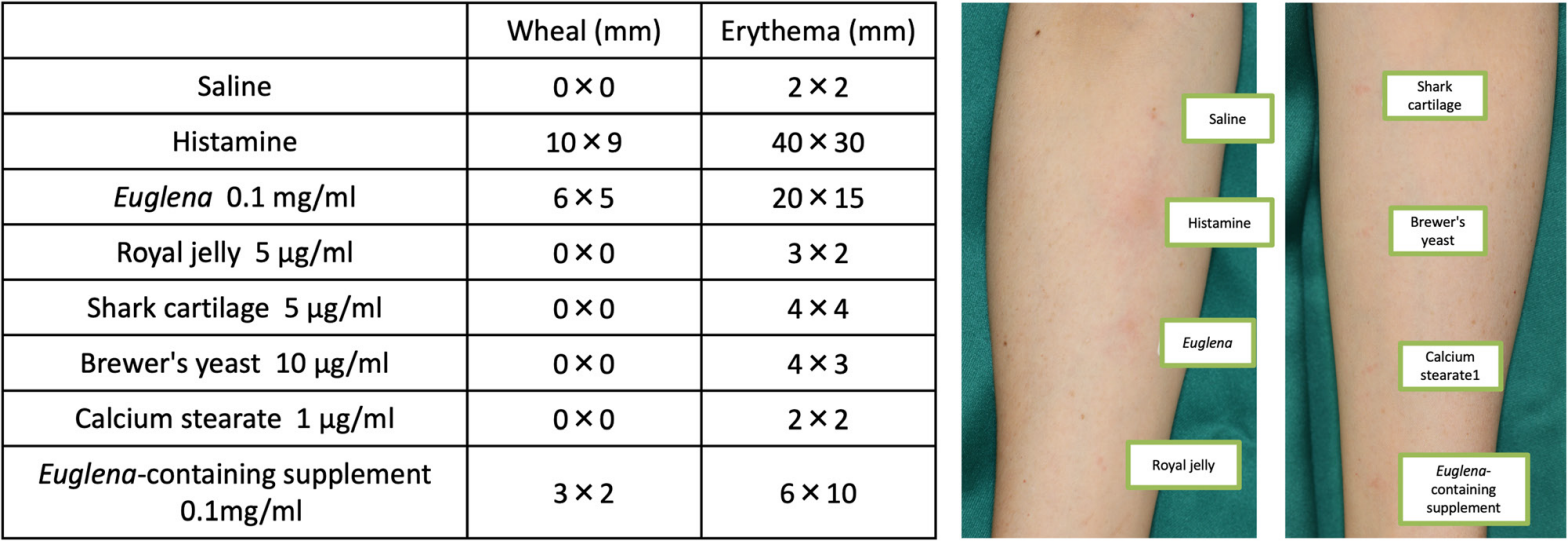
Skin-prick test and western blot analysis for Euglena. Skin-prick test using *Euglena*-containing supplements and raw materials. A positive reaction was observed with *Euglena* and a weak positive reaction with the *Euglena*-containing supplement. Testing on healthy controls showed no positive reactions.

HRT using the *Euglena*-containing supplement demonstrated a dose-dependent histamine release from basophils (Fig. 1A). HRT was also conducted with the individual components of the supplement, including *Euglena*, royal jelly, shark cartilage, brewer’s yeast, and calcium stearate. Only *Euglena* showed concentration-dependent histamine release from basophils (Fig. 1B), while all other components resulted in histamine release levels of nearly 10%, regardless of concentration (data not shown). Based on the HRT results, skin-prick tests were performed, which yielded a positive response to *Euglena*. A stronger reaction was observed with the *Euglena*-containing supplement than with the negative control, although it did not meet the criteria for a positive result and was thus classified as weakly positive (Fig. 2).

Based on these findings, the patient was diagnosed with *Euglena*-induced anaphylaxis and advised to avoid further *Euglena* intake. No oral challenge test was performed. After discontinuing the *Euglena*-containing supplement, the patient did not experience any recurrence of urticaria or anaphylactic symptoms.

## 3. Discussion

A previous case of an allergic reaction to *Euglena* has been documented, presenting as erythema multiforme-like eruptions [2]. Based on the clinical course and results of the lymphocyte transformation test (LTT), a delayed-type allergy was suspected; however, LTT verification did not confirm it as the definitive cause. In contrast, our patient was diagnosed with an immediate allergic reaction to *Euglena*, specifically attributed to the *Euglena*-containing supplement. This diagnosis was supported by the results of both HRT and skin-prick tests, along with the resolution of allergic symptoms following discontinuation of the supplement.

It is speculated that the patient may have become sensitized to Euglena over the 10 years of taking small amounts of supplements and that increasing the amount of intake over the past 3 months exceeded the antigen dose threshold, causing anaphylaxis.

## 4. Conclusion

Here, we report a case of anaphylaxis following prolonged intake of an *Euglena*-containing supplement. To the best of our knowledge, this is the first documented instance of an immediate allergic reaction triggered by *Euglena.* As the consumption of *Euglena*-containing products continues to grow, there may be an increase in cases of allergic reactions to *Euglena*.

## Conflicts of interest

The authors declare no conflict of interest.

## Author contributions

Murakami identified and managed the clinical case, conducted a literature review, and drafted the initial manuscript. Suehiro assisted with the skin testing procedures and contributed to revising the manuscript. Sasaki and Kamigaki assisted with both the skin testing procedures and histamine release tests. Tanaka provided expert guidance on diagnostic examinations and patient management. Ishii the lead author, was responsible for collecting histamine release test data and making critical revisions to the manuscript. All authors reviewed and approved the final version of the manuscript.
